# Transcriptomic and metabolomic profiles of Chinese citrus fly, *Bactrocera minax* (Diptera: Tephritidae), along with pupal development provide insight into diapause program

**DOI:** 10.1371/journal.pone.0181033

**Published:** 2017-07-12

**Authors:** Jia Wang, Huan Fan, Ke-Cai Xiong, Ying-Hong Liu

**Affiliations:** Institute of Entomology, College of Plant Protection, Southwest University, Chongqing, China; USDA Agricultural Research Service, UNITED STATES

## Abstract

The Chinese citrus fly, *Bactrocera minax* (Enderlein), is a devastating citrus pest in Asia. This univoltine insect enters obligatory pupal diapause in each generation, while little is known about the course and the molecular mechanisms of diapause. In this study, the course of diapause was determined by measuring the respiratory rate throughout the pupal stage. In addition, the variation of transcriptomic and metabolomic profiles of pupae at five developmental stages (pre-, early-, middle-, late-, and post-diapause) were evaluated by next-generation sequencing technology and ^1^H nuclear magnetic resonance spectroscopy (NMR), respectively. A total of 4,808 genes were significantly altered in ten pairwise comparisons, representing major shifts in metabolism and signal transduction as well as endocrine system and digestive system. Gene expression profiles were validated by qRT-PCR analysis. In addition, 48 metabolites were identified and quantified by ^1^H NMR. Nine of which significantly contributed to the variation in the metabolomic profiles, especially proline and trehalose. Moreover, the samples collected within diapause maintenance (early-, middle-, and late-diapause) only exhibited marginal transcriptomic and metabolomic variation with each other. These findings greatly improve our understanding of *B*. *minax* diapause and lay the foundation for further pertinent studies.

## Introduction

The Chinese citrus fly, *Bactrocera minax* (Enderlein), is a devastating oligophagous pest of citrus plants in the temperate areas of Asia, especially in China [1–3]. Larval feeding can cause serious yield losses [4–6], as such this pest has become a focus of interest in citrus-growing regions in China. Given the economic importance of *B*. *minax*, a number of prior studies have been carried out [7–13]. However, the long-lasting pupal stage in which diapause occurs has severely hindered the laboratory-rearing of this pest and restricted further scientific research.

Diapause is a life history stage that allows insects to mitigate acute environmental stresses [14,15]. Some univoltine insects enter obligatory diapause at specific stages in each generation, but require no token stimuli for diapause induction and preparation [14,16]. Similarly, univoltine tephritid fly *B*. *minax* also enters the pupal diapause to overwinter. However, the course of diapause has yet to be reported. Generally, diapause in insects features intense metabolic depression. Respiratory rate is therefore a useful marker for determining diapause initiation and termination [17]. For example, the precise time of diapause termination of *Rhagoletis pomonella* was identified based on the fitted trajectory of metabolic rate estimated by CO_2_ production. Subsequently, samples during the diapause termination were collected to investigate the molecular mechanisms underlying diapause termination and resumption of development [16]. By determining the course of diapause in *B*. *minax* through respiratory rate measurement, several other investigations into diapause can be conducted.

Next-generation sequencing has widely been used to characterize genomes and transcriptomes. This approach has facilitated studies on biological processes in organisms, such as the discovery of differentially expressed genes (DEGs) [18–20]. A *B*. *minax* transcriptome that was previously assembled and annotated can provide a foundation for further DEG analysis [21]. Similarly, metabolomic profiling has increasingly been used worldwide to investigate the qualitative and quantitative variation of metabolites in tissues and biofluids in response to biotic or abiotic factors [22]. The most widely used methods for metabolomic analysis are ^1^H nuclear magnetic resonance spectroscopy (NMR), gas-chromatography coupled with mass spectrometry (GC-MS), and liquid chromatography coupled with mass spectrometry (LC-MS) [23,24]. Of these methods, NMR has significant advantages, including near-universal detection of metabolites, easy quantitation, high reproducibility, minimal sample preparation, and rapidity [25,26]. So far, metabolomic analysis has been performed in many fields such as diagnostics [27], pharmacology [28], microbiology [29], and nutrition [30]. Recently, a limited number of metabolomic analyses have also been conducted on insect diapause [31–33].

In this study, the respiratory rate of *B*. *minax* was periodically measured throughout the pupal stage in order to clarify the course of diapause. To better understand the molecular mechanisms underlying diapause, high-throughput next-generation sequencing technology and ^1^H NMR analysis were performed to analyze respectively the transcriptomic and metabolomic profiles of *B*. *minax* at five pupal developmental stages. The results have revealed variations in the physiological pathways utilized throughout the course of diapause and provided new insights into diapause programming.

## Materials and methods

### Ethics statement

The owner of the orchard in Wulong County, Chongqing Municipality, China, provided permission to collect the samples for our scientific research.

### Insect rearing

Fallen oranges infested with larvae were brought back to the lab from an orchard (N 29^o^ 34.373', E 107^o^ 54.564') in Wulong County, Chongqing Municipality, China. Third-instar larvae (15~18 mm in length) collected from the oranges were placed over sand in plastic dishes and allowed to pupate. All dishes were placed outdoors in the nylon insect rearing cage under natural temperature and light/dark cycle in the Beibei district, Chongqing Municipality, China. The sand was changed weekly and regularly watered to maintain moisture.

### Measurement of respiratory rate of pupae

The respiratory rate of each group was measured 46 times from pupation to the first adult emergence, using a self-designed portable respirometry system with a high-accuracy infrared CO_2_ detector (S1 Fig Wosaite, Shenzhen, China). Prior to measuring the pupal respiratory rate, thirty pupae were randomly selected and divided into three groups. Each group was weighed and placed in a 50 mL sealed chamber for 2 h and the CO_2_ concentration in the chamber was recorded. The respiratory rate of pupae was calculated by:

$$R=\frac{C.V}{M.T}$$ (1)

Where *R* is the calculated respiratory rate in μL CO_2_/mg/h, *C* is the raised CO_2_ concentration in μL/L, *V* is the volume of the sealed chamber in L, *M* is the weight of the pupae in mg, and *T* is the duration of CO_2_ measurement in h. Visual inspection of respiratory rate data strongly suggested an exponential decay followed by a logistic increase and an exponential increase, based on which an eight parameter non-linear model describing this trajectory was constructed:

$$R=(\alpha -\beta )e^{-kt}+\frac{\gamma }{1+e^{\left(\alpha -rt\right)}}+ce^{bt}+\beta$$ (2)

Where *R* is the respiratory rate, *t* is the time in days since investigation, *α* is the respiratory rate at the starting point, *β* is the respiratory rate at the transition between exponential decay and logistic increase, *k* is the rate constant, *γ* is the respiratory rate at the transition between logistic and exponential increase, *a* is the constant, *c* is the scaling parameter, *r* and *b* are parameters that determine the timing of two transitions, respectively. The non-linear model was fitted using GraphPad Prism 5 and Microsoft Excel 2010, and the goodness of fit was determined by χ^2^ test using Excel 2010. The initiation, maintenance, and termination of pupal diapause were determined in relation to the fitted model.

### Insect sample collection

**Fig 1:**
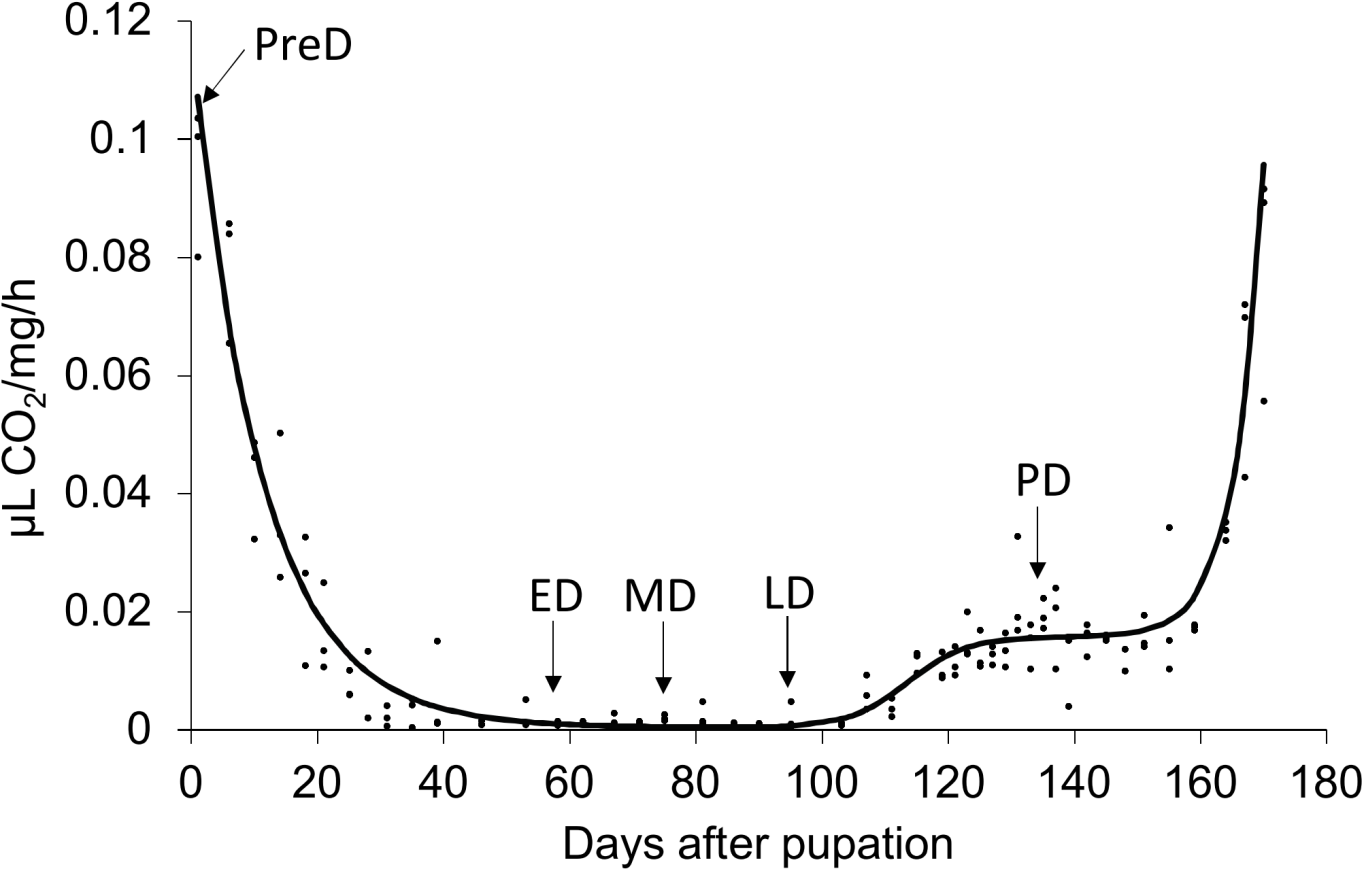
Non-linear regression of respiratory rate of *Bactrocera minax* pupae. Arrows indicate the time points when the samples were collected for transcriptomic and metabolomic analysis.

Samples were collected at five time points, pre-diapause (PreD), early-diapause (ED), middle-diapause (MD), late-diapause (LD), and post-diapause (PD) (Fig 1), as determined by respiratory rate. Prior to sample collection, all pupae were reared separately in 15 dishes, three dishes for each of the five developmental stages, under the conditions described above. At each sampling point, all of the pupae in the four dishes were collected and stored separately in liquid nitrogen for subsequent transcriptomic and metabolomic analysis.

### RNA isolation, library construction and Illumina sequencing

Total RNA from each pupa was isolated using TRIZOL Reagent (Life technologies, Carlsbad, CA, US) according to the manufacturer's instructions. The quantity and quality of total RNA was assessed with a NanoVue spectrophotometer (GE Healthcare Bio-Science, Uppsala, Sweden) and 1% agarose gel electrophoresis, respectively. The cDNA libraries were constructed using TruSeq RNA Sample Preparation Kit (Illumina, San Diego, CA, USA) following the manufacturer’s protocol. Briefly, oligo (dT) magnetic beads were used to purify poly (A) mRNA. The resulting mRNA was mixed with fragmentation buffer and cleaved into short fragments. The first-strand cDNAs were generated with random hexamer primers. Second-strand cDNAs were synthesized using DNA polymerase I (New England BioLabs, Ipswich, MA) and RNase H (Invitrogen, Carlsbad, CA). These cDNA fragments were end-repaired, followed by single nucleotide A (adenine) addition and litigation of adaptors. After purification with AMPureXP beads, the ligated products were amplified by PCR to generate cDNA libraries, which were sequenced on an Illumina NextSeq500 (Illumina). The raw reads were filtered to remove adaptor sequences, low-quality sequences with unknown nucleotides N, reads shorter than 50 bp, and reads with more than 20% low quality bases, using the NGS QC toolkit package [34]. The clean reads were assessed for quality using FastQC (http://www.bioinformatiH2.babraham.ac.uk/projects/fastqc/) and then mapped to our previously generated transcriptome reference database [21]. Three biological replicates were generated for each developmental stage.

### Discovery of differentially expressed genes and KEGG pathway analysis

Gene expression levels were determined by reads per kb of exon model per million mapped reads (RPKM) values [35], which were calculated based on the number of reads mapping to each unigene obtained previously [21]. The DEseq package was used to identify the DEGs [36]. Unigenes with *P* value < 0.05 and fold change value > 2 in each comparison were considered to be significantly differentially expressed genes. Benjamini-Hochberg correction was then conducted to reduce the false discovery rate (FDR). DEG cluster analysis was performed using cluster [37] and visualized by Java Tree view [38]. Principal component analysis (PCA) was carried out using pcaMethods [39] to evaluate the variation in gene expression profiles among samples and visualize the clustering of samples. Kyoto Encyclopedia of Genes and Genomes (KEGG) pathways enrichment analyses were conducted using KEGG mapper to categorize the DEGs (http://www.genome.jp/kegg/tool/map_pathway2.html), and the *P* value < 0.01 was set as a threshold to determine the significantly regulated pathway in each comparison.

### Quantitative real-time PCR (qRT-PCR) verification

qRT-PCR was performed to verify the accuracy of the DEG analysis. Total RNA from the five pupal stages described above was extracted using TRIZOL Reagent. The first-strand cDNA was synthesized using PrimeScript™ RT Master Mix (Perfect Real Time) Kit (Takara, Shiga, Japan). Twenty-one pairs of specific primers were designed to amplify the genes selected from multiple comparison (S1 Table). *Ubiquitin* was used as a reference gene for normalization [40]. qRT-PCR was conducted in 25 μL volumes containing 12.5 μL SYBR^®^ Premix Ex Taq II (Takara), 2 μL primers (10 μM), 1μL cDNA, and 9.5 μL ddH_2_O, using a CFX96™ Real-Time PCR Detection System thermal cycler (BIO-RAD, Hercules, CA, USA). Amplification conditions were as follows: initial denaturation at 95°C for 30s; followed by 40 cycles of denaturation at 95°C for 5s, 60°C for 30s. Pearson’s r correlation coefficient was calculated to evaluate the correlation between the qRT-PCR and DEG data. Three biological and three technical replicates were performed for each gene.

### ^1^H NMR spectroscopy

About 500 mg pupae were ground in liquid nitrogen and lyophilized in a vacuum freeze dryer. More than 50 mg lyophilized sample was weighed and resuspended in 1 ml water. After vortexing for 1 min, samples were sonicated and centrifuged at 13000 rpm at 4°C for 10 min. The supernatant was filtered using 3-kDa microcentrifuge filters to remove proteins and insoluble impurities. A 450 μL filtrate was then mixed with 50 μL DSS standard solution (4.088 mM), an internal NMR chemical shift standard, for subsequent NMR analysis. One-dimensional NMR spectra of samples were acquired using a Bruker AV III 600 MHz spectrometer (Bruker Biospin Ltd., Coventry, UK) equipped with an inverse cryoprobe, operating at an NMR frequency of 600.13 MHz, and a data acquisition temperature of 298 K. A total of 64 transients were acquired in 32,768 data points using a spectral width of 8000 Hz. The free induction decay (FID) signal was multiplied by an exponential window function with 1 Hz line broadening factor before Fourier transform. Metabolites were assigned by Chenomx (Chenomx Inc., Edmonton, Canada) on the basis of chemical shifts, coupling constants, and relative intensities, against a Chenomx database that contained the unique NMR spectra of each standard compound. The absolute concentration of each compound was normalized to the sample weight. Five biological replicates were set for each selected developmental stage. PCA and partial-least squares discriminant analysis (PLS-DA) [41] were carried out to evaluate the variation in metabolite profiles among developmental stages and to visualize sample clustering. Based on PLS-DA analysis, the Variable Importance in Projection (VIP) scores were obtained to indicate the metabolites that significantly contributed to the intergroup differentiation. The concentrations of nine identified metabolites (VIP score > 1) at five developmental stages were compared using a non-parametric Kruskal-Wallis test, followed by Bonferroni correction [42]. The analyses were performed using the statistical package STATISTICA version 10 (StatSoft Inc. 2011, Tulsa, OK).

## Results

### Respiratory rate trajectory

The model describing the respiratory rate trajectory was fitted well according to Chi-square test (*χ*^*2*^ = 0.0824, *d*.*f*. = 44, *P* > 0.05). The exponential decay, logistic increase, and exponential increase in this model represent periods of diapause preparation, diapause termination, and preparation for adult emergence, respectively (Fig 1). According to this model, respiration was suppressed after pupation and reached its lowest level approximately 40 days later, when pupae had completely entered diapause. Approximately two months after initiation, diapause terminated and pupal development resumed with the respiratory rate increasing by 39-fold. Post-diapause development lasted for over a month until adults started to emerge.

### Identification of differentially expressed genes (DEGs) among the differing developmental stages

**Fig 2:**
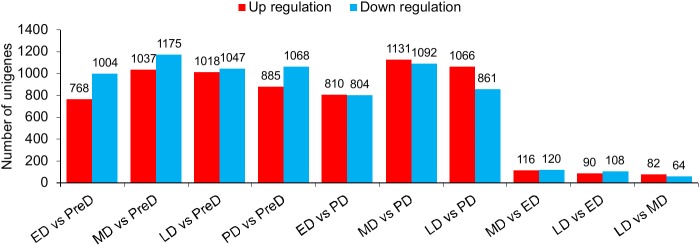
Number of significantly differentially expressed genes (DEGs) in each pairwise comparison of different *Bactrocera minax* pupal stages. PreD, pre-diapause. ED, early-diapause. MD, middle-diapause. LD, late-diapause. PD, post-diapause.

**Fig 3:**
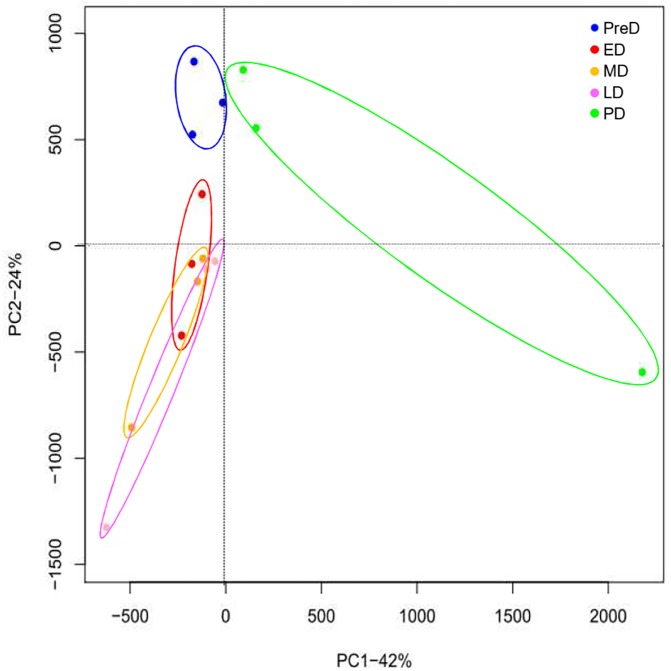
Principal component analysis (PCA) plot of gene expression profile at different *Bactrocera minax* pupal stages. PreD, pre-diapause. ED, early-diapause. MD, middle-diapause. LD, late-diapause. PD, post-diapause.

**Fig 4:**
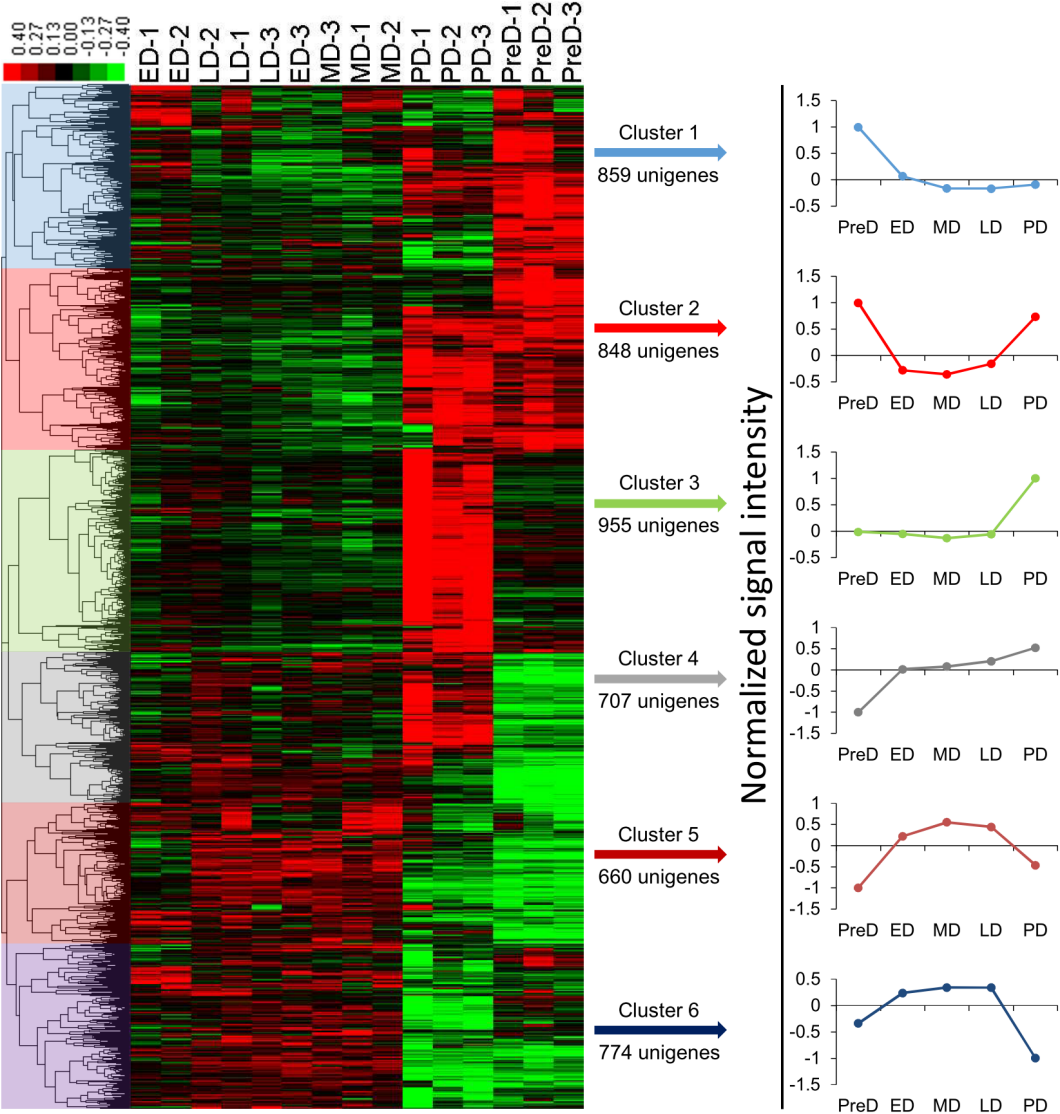
Cluster of differentially expressed genes (DEGs) among the different *Bactrocera minax* pupal stages. Each column represents a sample, and each row represents a differentially expressed gene. Green and red color gradients indicate a decrease or increase in expression, respectively. PreD, pre-diapause. ED, early-diapause. MD, middle-diapause. LD, late-diapause. PD, post-diapause.

Fifteen mRNA libraries, three replicates for each developmental stage, were sequenced. At least 18 million raw reads were generated in each library (S2 Table). All raw data have been deposited in the GeneBank Sequence Read Archive (Accession number: SRP083788). After removing low quality reads, the number of clean reads ranged from 18.37 million to 51.87 million, and the proportion of useful reads exceed 97% in all libraries. Gene expression profiles at different pupal developmental stages were calculated to identify DEGs. A total of 4,808 genes were significantly differentially expressed in ten pairwise comparisons (Fig 2 and S2 Fig), whereas the number decreased to 3,290 after Benjamini-Hochberg correction to reduce FDR (S3 Fig). Interestingly, samples collected within the maintenance of diapause (ED, MD, and LD) did not show intense variation in gene expression profiles compared to each other, with only a few genes altered. The large overlap of ED, MD, and LD in the PCA plot also showed no obvious difference in gene expression profiles among these three stages (Fig 3). However, a distinct separation in the PCA plot and a number of DEGs between PreD/PD and the other time points revealed large variations in the gene expression profiles between these two stages and others (Figs 2 and 3). All DEGs were divided into 6 groups with each exhibiting a representative expression pattern. Genes in cluster 1 and 3 were highly expressed in PreD and PD, respectively, whereas genes in cluster 4 were lowly expressed in PreD. Throughout diapause maintenance, the expression of genes in cluster 2 was suppressed, whereas those in cluster 5 and 6 were activated (Fig 4).

To understand the potential functions of identified DEGs, KEGG pathway enrichment for each pairwise comparison was performed at *P* value < 0.01. Pathways involved in human disease were excluded after analysis. The results showed that no KEGG pathway was enriched in pairwise comparisons among the three groups from maintenance of diapause, except for “Alanine, aspartate, and glutamate metabolism” between MD and LD. Most of the significantly altered pathways in other comparisons were related to “Carbohydrate metabolism”, “Lipid metabolism”, “Amino acid metabolism”, “Biosynthesis of other secondary metabolites”, “Metabolism of cofactors and vitamins”, “Xenobiotics biodegradation and metabolism”, “Signal transduction”, “Endocrine system”, and “Digestive system” (Table 1).

**Table 1:** KEGG pathway analysis of differentially expressed genes among comparisons.

Comparison	KEGG pathway	Comparison	KEGG pathway
ED *vs* PreD	Pentose and glucuronate interconversions*	PD *vs* PreD	Pentose and glucuronate interconversions*
	Galactose metabolism*		Galactose metabolism
	Ascorbate and aldarate metabolism		Ascorbate and aldarate metabolism*
	Starch and sucrose metabolism*		Starch and sucrose metabolism
	Glycerolipid metabolism		Steroid hormone biosynthesis
	Ether lipid metabolism		Glycerolipid metabolism
	Arginine and proline metabolism		Retinol metabolism
	Glutathione metabolism*		Metabolism of xenobiotics by cytochrome P450*
	Retinol metabolism		Drug metabolism—cytochrome P450*
	Porphyrin and chlorophyll metabolism		DNA replication
	Metabolism of xenobiotics by cytochrome P450*		Cell adhesion molecules (CAMs)
	Drug metabolism—cytochrome P450*		PPAR signaling pathway*
	Protein processing in endoplasmic reticulum		Protein digestion and absorption
	Antigen processing and presentation	ED *vs* PD	Biosynthesis of amino acids*
	Estrogen signaling pathway		Ascorbate and aldarate metabolism
	Protein digestion and absorption		Starch and sucrose metabolism*
	Fat digestion and absorption		Glycine, serine and threonine metabolism*
MD *vs* PreD	Biosynthesis of amino acids*		Retinol metabolism
	Pentose and glucuronate interconversions*		Porphyrin and chlorophyll metabolism
	Galactose metabolism		Antigen processing and presentation
	Starch and sucrose metabolism*	MD *vs* PD	Carbon metabolism
	Amino sugar and nucleotide sugar metabolism		Biosynthesis of amino acids*
	Nitrogen metabolism		Pentose phosphate pathway*
	Fatty acid degradation		Ascorbate and aldarate metabolism
	Glycerolipid metabolism		Starch and sucrose metabolism
	Glycerophospholipid metabolism		Steroid hormone biosynthesis
	Alanine, aspartate and glutamate metabolism*		Glycine, serine and threonine metabolism
	Arginine and proline metabolism*		Glutathione metabolism
	Glutathione metabolism		Retinol metabolism
	Streptomycin biosynthesis		Porphyrin and chlorophyll metabolism
	Aminobenzoate degradation		Metabolism of xenobiotics by cytochrome P450
	Two-component system		Drug metabolism—cytochrome P450
	Hippo signaling pathway -fly		MAPK signaling pathway
	Peroxisome		Hippo signaling pathway -fly
	Ovarian Steroidogenesis		Cell adhesion molecules (CAMs)
	Estrogen signaling pathway		Antigen processing and presentation
	Fat digestion and absorption*	LD *vs* PD	Purine metabolism*
LD *vs* PreD	Pentose and glucuronate interconversions*		Glutathione metabolism
	Galactose metabolism*		Wnt signaling pathway
	Starch and sucrose metabolism*		Hippo signaling pathway -fly
	Nitrogen metabolism		Insulin secretion
	Fatty acid degradation		Adrenergic signaling in cardiomyocytes
	Glycerolipid metabolism*		Pancreatic secretion
	Glycerophospholipid metabolism*	MD vs LD	Alanine, aspartate and glutamate metabolism
	Glycosaminoglycan degradation		
	One carbon pool by folate		
	Retinol metabolism		
	Caffeine metabolism		
	Drug metabolism—other enzymes		
	Neuroactive ligand-receptor interaction		
	Peroxisome*		
	PPAR signaling pathway*		
	Protein digestion and absorption		
	Fat digestion and absorption		

* *P* value < 0.001. For KEGG pathways without asterisks, *P* value < 0.01.

### Validation of gene expression profiles

**Fig 5:**
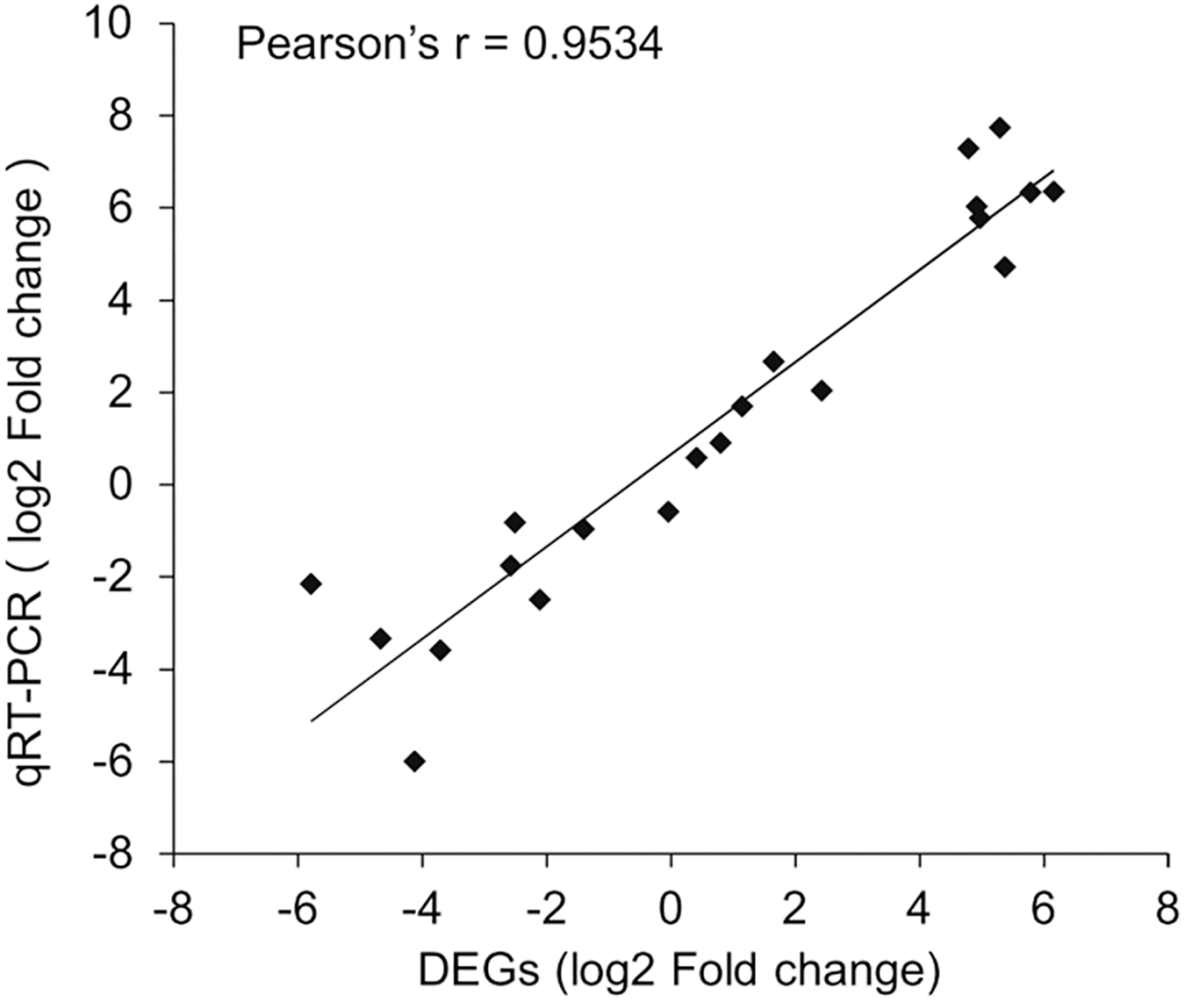
Correlation analysis of qRT-PCR and differentially expressed gene (DEG) data for selected genes of *Bactrocera minax*.

The expression levels of the 21 selected genes were measured by qRT-PCR to validate the DEG analysis. The results showed a strong correlation between the qRT-PCR and DEG data with Pearson’s correlation coefficient > 0.95 (Fig 5), indicating the reliability of using DEG data to investigate temporal-specific gene expression profiles throughout the pupal stage.

### Metabolomic variation among *Bactrocera minax* developmental stages

**Fig 6:**
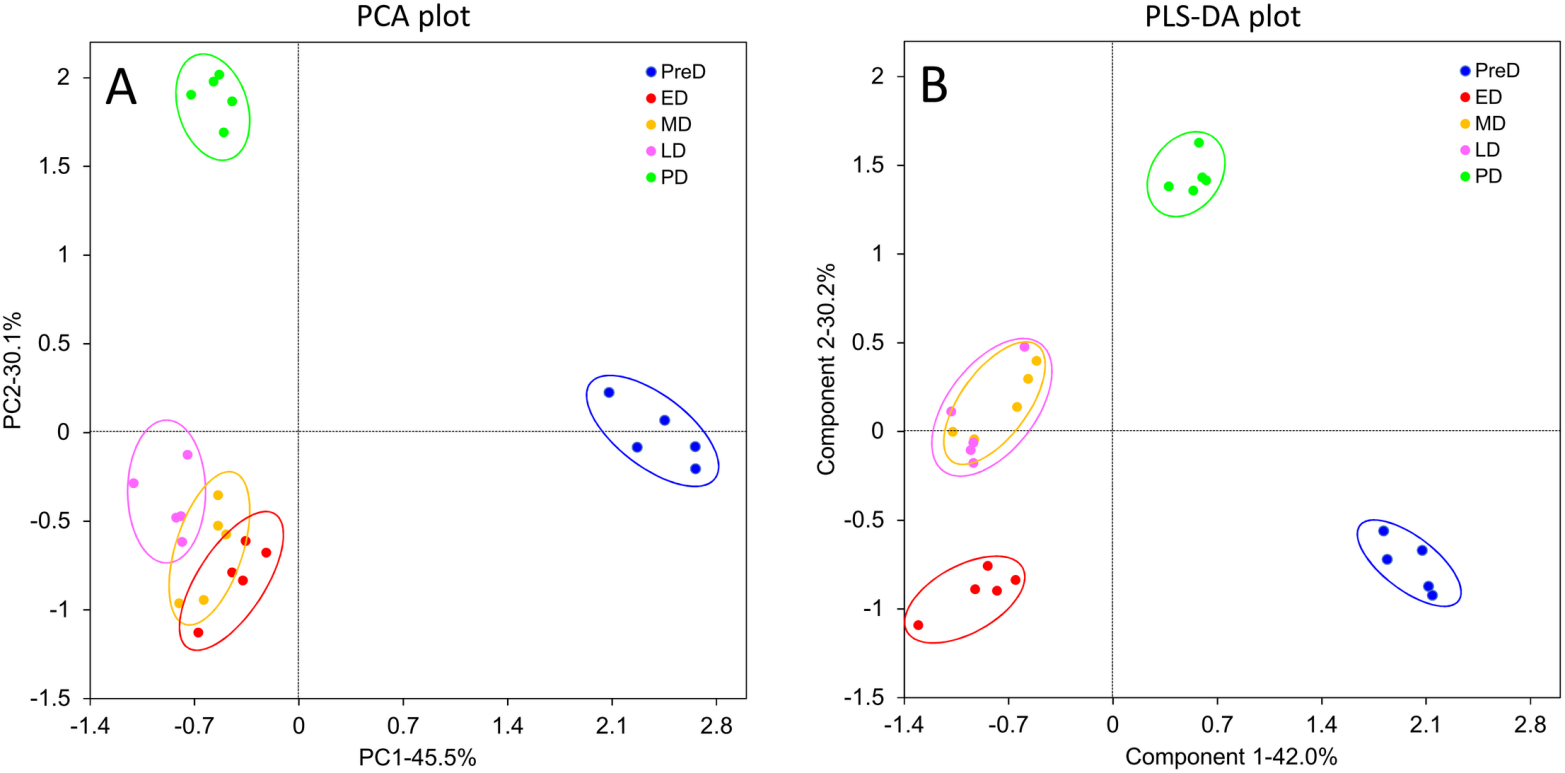
**Principal component analysis (PCA) plot (A) and partial-least squares discriminant analysis (PLS-DA) plot (B) of metabolite profiles from different *Bactrocera minax* pupal stages.** PreD, pre-diapause. ED, early-diapause. MD, middle-diapause. LD, late-diapause. PD, post-diapause.

**Fig 7:**
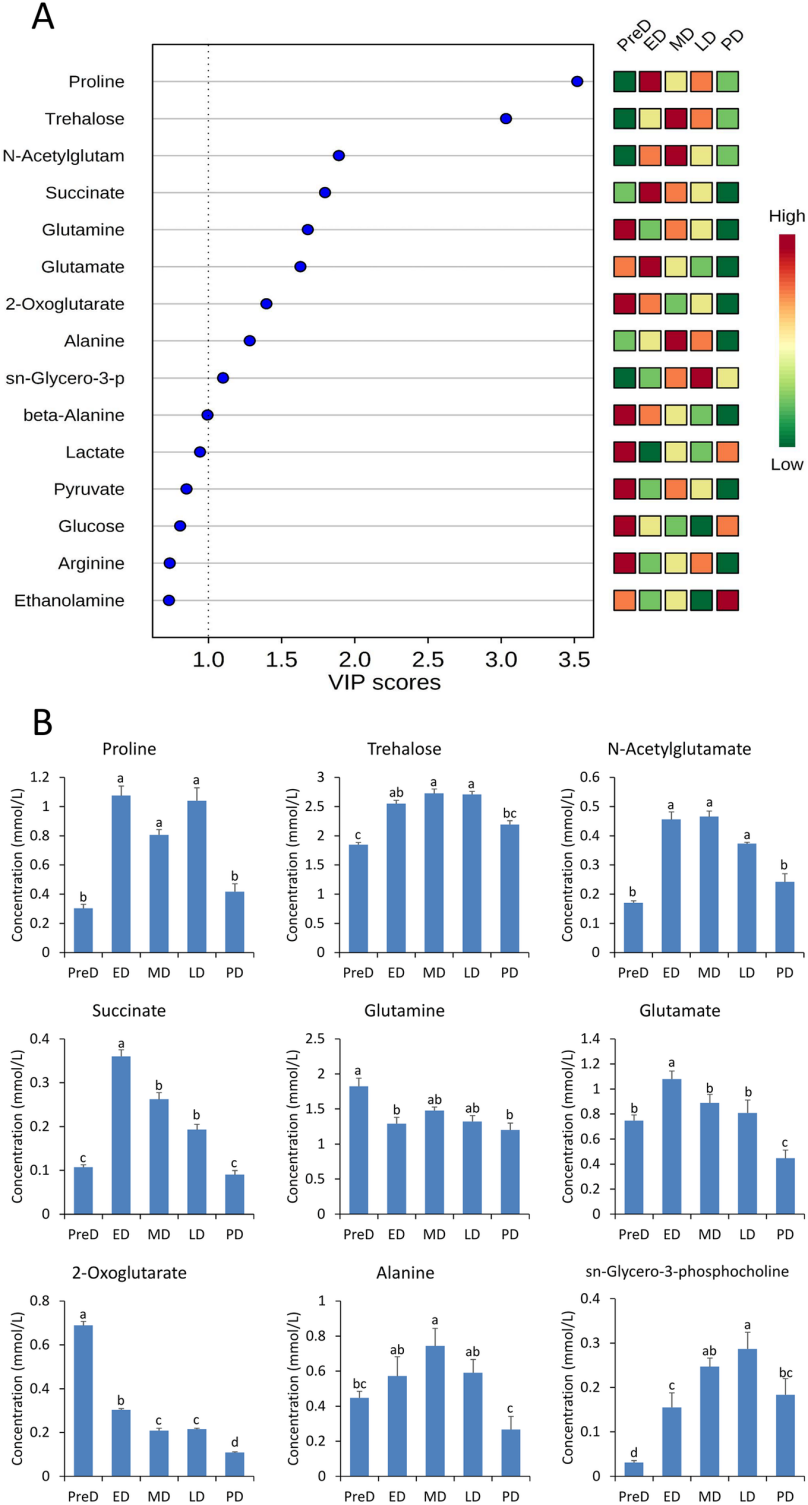
Metabolomic variation among different *Bactrocera minax* pupal stages. A. Variable importance plot showing the metabolites with the highest VIP score. B. Concentration of nine metabolites (VIP score >1) at different *B*. *minax* pupal stages. Different letters above the bars indicate significant differences determined by Bonferroni correction. PreD, pre-diapause. ED, early-diapause. MD, middle-diapause. LD, late-diapause. PD, post-diapause.

A total of 49 metabolites were identified across all samples, including amino acids and their derivatives, organic acids, nucleic acid components, sugars, and others (S4 Fig). After removing citrate, which cannot be precisely quantified due to the effect of pH value, the other 48 metabolite identities were confirmed for subsequent analysis. Both the PCA and PLS-DA plots showed close overlap between the ED, MD, and LD groups, but also showed clear separation between PreD and PD (Fig 6), indicating the metabolomic profiles changed marginally within diapause maintenance, but varied dramatically with initiation and termination of diapause. After VIP score calculation, the top 9 metabolites that contributed the most to the variation of metabolomic profiles (VIP score > 1) were identified (Fig 7A). Significant differences were found in the concentration of nine metabolites across the five developmental stages with non-parametric Krustal-Wallis test (Table 2). Of all these nine metabolites, proline, trehalose, N-acetylglutamate, succinate, glutamate, alanine, and sn-glycero-3-phosphocholine saw significant accumulations within maintenance of diapause; the glutamine concentration was higher in PreD; and the 2-oxoglutarate concentration was higher in PreD but gradually decreased along with development (Fig 7B).

**Table 2:** Identification of significantly altered metabolites with non-parametric Krustal-Wallis test.

Common name	Chemical shift (ppm)	Formula	d.f.	n	H value	*P*-value
Proline	2.00 (m)	C_5_H_9_NO_2_	4	25	20.11	<0.01
Trehalose	3.44(t), 3.80(m), 5.18(d)	C_12_H_22_O_11_	4	25	19.64	<0.01
N-Acetylglutamate	2.02(s)	C_7_H_11_NO_5_	4	25	21.84	<0.01
Succinate	2.39(s)	C_4_H_6_O_4_	4	25	22.00	<0.01
Glutamine	2.11(m)	C_5_H_10_N_2_O_3_	4	25	13.23	<0.01
Glutamate	2.04(m), 2.34(m)	C_5_H_9_NO_4_	4	25	21.00	<0.01
2-Oxoglutarate	2.99(t)	C_5_H_6_O_5_	4	25	21.94	<0.01
Alanine	1.46(d)	C_3_H_7_NO_2_	4	25	18.88	<0.01
Sn-Glycero-3-phosphocholine	4.31(m)	C_8_H_21_NO_6_P	4	25	21.61	<0.01

## Discussion

### Respiratory rate trajectory

Metabolic depression is frequently considered a universal characteristic of diapausing insects [16,17,43], hence, the respiratory rate of *B*. *minax* pupae was measured first to determine the course of diapause. The sensitivity of the CO_2_ detector precluded monitoring respiratory rate of individual *B*. *minax*. However, monitoring a group of pupae proved to be feasible as all adults emerged within 8 days (data not shown), indicating synchronization of pupal development. The generated curve identified important developmental landmarks throughout the pupal stage and guided our sample collection (Fig 1). To our knowledge, this is the first endeavor to describe the course of diapause in *B*. *minax* pupae, which provided the fundamental basis for subsequent transcriptomic and metabolomic analyses in this study.

### Gene expression profiles throughout pupal development

Gene expression profiles varied within pupal development, but not much within the maintenance of diapause (Figs 2 and 3). When comparing PreD with others, the most significantly changed KEGG pathways were associated with metabolism, especially the “Pentose and glucuronate interconversions”, “Galactose metabolism”, “Starch and sucrose metabolism”, and “Glycerolipid metabolism” pathways, which were significantly changed in all four comparisons. These variations were consistent with the suppression in respiratory rate described above. When comparing PD with ED/MD, several metabolism-related KEGG pathways significantly changed, probably due to elevation in the metabolic rate after diapause termination. However, when comparing PD with LD, most of the enriched KEGG pathways related to signaling pathways. The “Wnt signaling pathway” and “Hippo signaling pathway—fly” is expected to be responsible for cell proliferation and differentiation in post-diapause development [44,45]. The “insulin secretion” pathway may also be involved in the insulin signaling pathway which is a conserved mechanism for controlling insect diapause [46,47].

To survive the various biotic and abiotic stresses during diapause, a series of stress-induced mechanisms in insects have to be deployed. Heat shock proteins (Hsps) are a group of well-described proteins that play vital roles in cold hardiness and normal development during diapause [48,49]. Two Hsc70s, Hsc70-1 (BmUnigene24370.co) and Hsc70-4, (BmUnigene15892.co) were found up-regulated throughout diapause in this study (S5 Fig). Hsc70s are constitutively expressed members of the Hsp70 family and function as chaperone proteins that facilitate several biological processes including signal transduction, apoptosis, protein homeostasis, and cell growth and differentiation [50]. However, their exact roles in *B*. *minax* diapause remain unknown. Diapausing insects are commonly subjected to hypoxia or anoxia which may affect the intracellular redox potential [51]. To protect cells from oxidative damages predominantly caused by reactive oxygen species (ROSs), several enzymatic mechanisms may be activated, such as glutathione S-transferase (GST), superoxide dismutase (SOD), catalase (CAT), and peroxidases (POD) [52–55]. In *B*. *minax*, a microsomal GST (mGST, BmUnigene15924.co) and a Mn-SOD (BmUnigene23806.co) were up-regulated throughout diapause (S5 Fig). Likewise, members of the GST or SOD family were also up-regulated in other insects during diapause [32,56,57]. The up-regulation of these antioxidant proteins is speculated to protect individuals from oxidative damage in hypoxia/anoxia environments. Moreover, the expression of ryanodine receptor (RyR, BmUnigene27638.co) was higher in diapause (S5 Fig). The RyR protein functions as the major component of a calcium channel that mediates the release of calcium ions from the sarco/endoplasmic reticulum [58]. Calcium is an important second messenger, and its release from intracellular stores is critical for activation of many Ca^2+^-dependent pathways [59]. It has been demonstrated that intracellular Ca^2+^ is required for basal cellular metabolism and cell survival [60], and rendered cold tolerance to organisms [61]. This may also be the case for diapausing *B*. *minax*. These proteins potentially contribute to development and stress resistance during diapause, though the exact roles they play remain unknown.

The transcriptomic profile of *B*. *minax* pupal development investigated previously [8] is partially consistent with the current study. For example, both studies demonstrated that both metabolism and hormone biosynthesis were suppressed in diapause and several HSPs exhibited a diverse expression pattern during the pupal stage. However, crucial differences between the two studies exist, most likely due to the different periods of sampling and methods of sequencing.

### Metabolomic profile during pupal development

PCA and PLS-DA plots showed that the variation in metabolomic profiles throughout pupal development was consistent with that observed in the gene expression profiles (Fig 6). Based on VIP scores, the variation in the metabolomic profiles was mainly attributed to changes in nine metabolites (Fig 7 and Table 2), in particular proline and trehalose, which exhibited higher VIP scores than the others and were maintained at higher concentrations throughout diapause. Proline has been demonstrated to be an excellent cryoprotectant for insects to tolerate the formation of ice crystals in their body fluids [62]. Surprisingly, simply feeding *Drosophila melanogaster* larvae a proline-augmented diet can convert this chill susceptible insect to a freeze tolerant one, capable of surviving when 50% of its body water freezes [63]. Variations in the concentration of proline during pupal development were consistent with the DEG analysis which showed the “Arginine and proline metabolism” pathway was significantly altered (Table 1). Specifically, the up-regulation of two cytosol aminopeptidases (LAP3s, BmUnigene25466.co and BmUnigene21743.co) that release N-terminal proline from a peptide, and the down-regulation of three prolyl 4-hydroxylases (P4HAs, BmUnigene26736.c1, BmUnigene26352.co, and BmUnigene27483.co) that convert proline to hydroxyproline, may be conducive to proline accumulation throughout diapause (S5 Fig).

Trehalose is the principal sugar circulating in the hemolymph of most insects due to its unique chemical properties, and is an important agent for protecting cells from a variety of environmental stresses, such as dehydration, heat, freezing, and oxidation [64–66]. Trehalose accumulates in diapausing *B*. *minax* and many other winter diapausing insects, likely acting as a cryoprotectant to help organs resist temperature fluctuations [67–69]. Nevertheless, a trehalose 6-phosphate phosphatase (TPP, BmUnigene26329.co) that catalyzes the formation of trehalose was down-regulated (S5 Fig), and other TPPs were not significantly altered throughout diapause in this study. Therefore, the accumulation of trehalose may result from alterations in trehalases (Tres) that catalyze trehalose catabolism. Insect trehalase has been demonstrated to exist in two distinct forms, soluble (Tre-1) and membrane-bound (Tre-2) trehalase [70]. In the *B*. *minax* transcriptome database, only Tre-1 (BmUnigene24123.co) was identified and it did not show any significant alteration during the pupal stage (S5 Fig). Therefore, it can be inferred that another trehalase, Tre-2, may play an important role in the regulation of trehalose levels during pupal development. In addition, proline and trehalose are sources of energy in insects [64,71], accumulation of these two metabolites in diapause may facilitate energy-consuming post-diapause development.

N-acetylglutamate (NAcGlu) is an obligate activator of the urea cycle [72]. It has been reported that the accumulation of urea in diapause-destined larvae of cotton bollworm, *Helicoverpa armigera*, exerted a potential cryopreservative effect [32]. Thus, the elevated NAcGlu levels throughout pupal diapause may enhance urea production and contribute to cold resistance in *B*. *minax*. Moreover, the synthesis of NAcGlu by N-acetylglutamate synthase (NAGS) is stimulated by both arginine, an allosteric stimulator of NAGS, and glutamate, one of NAGS's substrates. Therefore, the high glutamate levels throughout diapause are expected to meet the requirement of NAcGlu synthesis. In this study, the elevated glutamate level may be associated with drastic down-regulation of two genes in the “Arginine, aspartate and glutamate metabolism” pathway (S5 Fig), glutamine synthetase (GlnA, BmUnigene25702.co) and glutamate dehydrogenase (GdhA, BmUnigene15155.co), which catalyze the conversion of glutamate to glutamine and 2-oxoglutarate, respectively.

Glutamine is a key metabolite playing roles in a variety of biochemical functions, such as protein and lipid synthesis, source of energy, nitrogen and carbon donation, and as a precursor to glutamate [73]. High concentrations of glutamine in PreD may be in preparation for relevant biological activities in diapause. During pupal development, the glutamine level is positively correlated to the expression level of GlnA, indicating the role of GlnA in the regulation of glutamine. Alanine is another amino acid that accumulated during diapause, which is consistent with several other studies [31,74,75]. High alanine levesl has been shown to exert synergistic and colligative effects with other solutes on cold tolerance [76].

Succinate and 2-oxoglutarate are intermediates in the tricarboxylic acid (TCA) cycle, which generates ATP and provides carbon skeletons for a range of biosynthetic processes. In diapausing *B*. *minax*, the TCA cycle may be inhibited as the concentrations of 2-oxoglutarate and two other intermediates identified in our study, pyruvate and fumarate, decreased. However, the concentrations of succinate increased in diapause, probably due to the up-regulation of succinyl-CoA synthetase (LSC1, BmUnigene28496.co) which converts succinyl-CoA to succinate and releases ATP/GTP by directly transferring a phosphoryl group to ADP/GDP (S5 Fig). This reaction is a substrate-level phosphorylation that provides a quicker but less efficient source of ATP/GTP, independent of external electron acceptors [77]. Therefore, the up-regulation of LSC1 is likely involved in maintaining the ATP/GTP levels under hypoxia and energy-limited conditions during diapause.

## Conclusion

In this study, transcriptomic and metabolomic analyses were performed to discover differentially expressed genes and significantly altered metabolites that coincided with pupal development. The findings provide insights into the diapause programming of *B*. *minax*. All DEGs and significantly altered metabolites identified may collectively play roles in stress resistance and survival of *B*.*minax* during diapause. However, the functions of most of the genes and metabolites remain unknown. To elucidate the mechanisms underlying pupal diapause of *B*. *minax* and comprehensively understand this pest, further investigations will focus on the specific aspects of biological variation along with pupal development or between diapausing and non-diapausing pupae.

## Supporting information

S1 Table Primer sequences used for qRT-PCR validation of DEGs. (DOC)

S2 Table Summary of Illumina sequencing data. (DOCX)

S1 Fig Schematic diagram of respirometry system with a high-accuracy infrared CO2 detector. (TIF)

S2 Fig Volcano plot of differentially expressed genes (DEGs) in each pairwise comparison of pupal stages of *Bactrocera minax*. Blue spot, expression of fold change > 2 and *P* value < 0.05. Orange spot, no difference in expression. PreD, pre-diapause. ED, early-diapause. MD, middle-diapause. LD, late-diapause. PD, post-diapause.
(TIF)

S3 Fig Number of significantly differentially expressed genes (DEGs) in each pairwise comparison of different *Bactrocera minax* pupal stages with Benjamini-Hochberg correction. PreD, pre-diapause. ED, early-diapause. MD, middle-diapause. LD, late-diapause. PD, post-diapause.
(TIF)

S4 Fig Representative ^1^H nuclear magnetic resonance (^1^H NMR) spectra of *Bactrocera minax* pupae. 1, 2-Hydroxybutyrate. 2, 2-Oxoglutarate. 3, 3-Aminoisobutyrate. 4, 3-Hydroxykynurenine. 5, Acetate. 6, Alanine. 7, Arginine. 8, Asparagine. 9, Aspartate. 10, Choline. 11, Citrate. 12, Cytidine. 13, Ethanol. 14, Ethanolamine. 15, Formate. 16, Fumarate. 17, Galactonate. 18, Glucose. 19, Glutamate. 20, Glutamine. 21, Glycine. 22, Guanosine. 23, Inosine. 24. Isoleucine. 25, Lactate. 26, Leucine. 27, Lysine. 28, Maltose. 29, Methanol. 30, N-Acetylglutamate. 31, O-Phosphocholine. 32, O-Phosphoethanolamine. 33, Pantothenate. 34, Phenylalanine. 35, Proline. 36, Pyruvate. 37, Succinate. 38, Threonate. 39, Threonine. 40, Trehalose. 41, Trigonelline. 42, Tryptophan. 43, Tyrosine. 44, UDP-N-Acetylglucosamine. 45, Uridine. 46, Valine. 47, sn-Glycero-3-phosphocholine. 48, trans-Aconitate. 49, β-Alanine. DSS, DSS Chemical Shape Indicator.
(TIF)

S5 Fig RPKM values of selected genes at different *Bactrocera minax* pupal stages. PreD, pre-diapause. ED, early-diapause. MD, middle-diapause. LD, late-diapause. PD, post-diapause. Hsc70, heat shock cognate protein 70. mGST, microsomal glutathione S transferase. Mn-SOD, Mn superoxide dismutase. RyR, ryanodine receptor. LAP3, cytosol aminopeptidases. P4HA, prolyl 4-hydroxylases. TPP, trehalose 6-phosphate phosphatase. Tre-1, trehalase-1. GlnA, glutamine synthetase. GdhA, glutamate dehydrogenase. LSC1, succinyl-CoA synthetase.
(TIF)
