# Supplementary material for: Transcriptomic and metabolomic profiles of Chinese citrus fly, Bactrocera minax (Diptera: Tephritidae), along with pupal development provide insight into diapause program
Source: PLoS One. 2017 Jul 12;12(7):e0181033. doi: 10.1371/journal.pone.0181033 (PMC5507520; doi:10.1371/journal.pone.0181033)
Supplement: S1 Table — (DOC) [file pone.0181033.s001.doc]

S1Table. Primer sequences used for qRT-PCR validation of DEGs.

| No. | Annotation | Primers | Comparison |
| --- | --- | --- | --- |
| 1 | lysyl oxidase homolog | AGCGCCGATTTAACTTTGGG | ED *vs* MD |
|  |  | TCCGCGTCTCCAACATTTTC |  |
| 2 | DNA-binding protein D-ETS-6-like | AACACTGAACAATGCGACCG | ED *vs* MD |
|  |  | GGTGCCTTCCCATGTTATGC |  |
| 3 | tubulin beta-1 chain-like | GTTCACTTGCAAGCTGGTCA | LD *vs* PD |
|  |  | ACTGCTGGCCTCATTGTAGT |  |
| 4 | chorion peroxidase-like | GCACGTACGGATGAAAGACC | LD *vs* PD |
|  |  | ATTCCTCCACCACCGACATT |  |
| 5 | xenotropic and polytropic retrovirus receptor | GCCCTTGGGTGAACAACAAA | LD *vs* PD |
|  |  | GAACGCCAGCCATACACATT |  |
| 6 | glutathione-s-transferase epsilon | CCACTTGTTAGCGTCGATGG | LD *vs* PD |
|  |  | ACGAAGCAGCCACATTGTTT |  |
| 7 | larval serum protein 1 beta chain-like | CGTGTGTAATCAACCGGGTG | ED *vs* LD |
|  |  | AGAACTGCTCGAAGTCCACA |  |
| 8 | phosphoglycolate phosphatase 1B | CGGTTACATAATCCGGCGTG | ED *vs* LD |
|  |  | GAATCCACGACGTTGCCTTT |  |
| 9 | DTW domain-containing protein 1-like | GCCATCTCAACATTGTGCCT | ED *vs* LD |
|  |  | CAGCAGTTTTGGCAAGGGAT |  |
| 10 | uncharacterized protein LOC101463445 | GGCCAAGCGTAAAGTGTCAA | ED *vs* LD |
|  |  | GACTGGGACGACACCTTACA |  |
| 11 | muscle-specific protein | ATGTTTGTAGGTGGCACGAC | PD *vs* ED |
|  |  | GTTCTGCGCTCTGATCAACA |  |
| 12 | protein sym1-like | GCCTTGAGTGCTCCAATACG | PreD *vs* ED |
|  |  | AAAATGGCGCCTCTTCGTTT |  |
| 13 | cytochrome P450 6a9-like | TCAGGCCCCGAAGATGAAAT | PreD *vs* ED |
|  |  | CTGGCTCTAGTAACGGCTGA |  |
| 14 | probable cytochrome P450 301a1, mitochondrial-like | TGTATGTGTGTGGCGGTAGT | PreD *vs* LD |
|  |  | AGAGACAGCAGAAACACGGA |  |
| 15 | angiopoietin-related protein 1-like | AGCTGCTGGTGGTGCTAATA | PreD *vs* LD |
|  |  | TAAGGACAAAGGGCGCCATA |  |
| 16 | WASH complex subunit FAM21 homolog isoform X1 | CAAGCGAATCTATAGCGGGC | PreD *vs* PD |
|  |  | AATATCGAACGCACCACAGC |  |
| 17 | uncharacterized protein LOC101461053 | CAAGGTCGGCGAGGTTATTG | PreD *vs* PD |
|  |  | CTGCTGCTAACGGAAAGGAC |  |
| 18 | probable peptidyl-tRNA hydrolase | CAGTTTGGTACGTGGTGCAG | PreD *vs* PD |
|  |  | TATCCGAAACAGGCGATTGG |  |
| 19 | H/ACA ribonucleoprotein complex subunit | TGACAACAGCAACAATGGCA | PreD *vs* PD |
|  |  | TTTGTGGTGCTACAGTTGCC |  |
| 20 | Drosophila pseudoobscura pseudoobscura | CGTTTTGGTTGCGAGGGTTA | PreD *vs* PD |
|  |  | GCAGCCGGGATGTTTAAGTC |  |
| 21 | uncharacterized protein | TTGACATGTGAAGTGCGTGG | PreD *vs* PD |
|  |  | GGTGTTGTTGAGAGCGGATC |  |
