# Supplementary material for: Transcriptomic and metabolomic profiles of Chinese citrus fly, Bactrocera minax (Diptera: Tephritidae), along with pupal development provide insight into diapause program
Source: PLoS One. 2017 Jul 12;12(7):e0181033. doi: 10.1371/journal.pone.0181033 (PMC5507520; doi:10.1371/journal.pone.0181033)
Supplement: S2 Table — (DOCX) [file pone.0181033.s002.docx]

S2 Table. Summary of Illumina sequencing data.

| **Sample** | **Reads** | **Raw Reads** | **Raw Data(bp)** | **GC(%)** | **Q20(%)** | **Clean Reads** | **Clean Data(bp)** | **Useful Reads(%)** | **Useful Data(%)** |
| --- | --- | --- | --- | --- | --- | --- | --- | --- | --- |
| ED-1 | Paired | 22,210,180 | 3,340,890,203 | 48.72% | 85.67% | 21,936,106 | 3,300,926,202 | 98.76% | 98.80% |
| ED-2 | Paired | 22,487,148 | 3,378,944,199 | 49.42% | 92.66% | 22,240,084 | 3,342,809,103 | 98.90% | 98.93% |
| ED-3 | Paired | 21,542,014 | 3,239,795,618 | 49.32% | 92.77% | 21,295,382 | 3,204,540,843 | 98.85% | 98.91% |
| LD-1 | Paired | 52,479,290 | 7,889,595,071 | 49.05% | 87.25% | 51,865,058 | 7,801,492,242 | 98.82% | 98.88% |
| LD-2 | Paired | 19,849,070 | 2,985,559,061 | 49.68% | 92.39% | 19,597,254 | 2,949,419,790 | 98.73% | 98.78% |
| LD-3 | Paired | 26,034,824 | 3,916,142,655 | 49.56% | 92.45% | 25,730,562 | 3,871,026,533 | 98.83% | 98.84% |
| MD-1 | Paired | 39,936,240 | 5,988,155,757 | 48.84% | 90.96% | 39,718,344 | 5,959,253,869 | 99.45% | 99.51% |
| MD-2 | Paired | 22,129,076 | 3,323,816,281 | 49.07% | 92.53% | 21,807,656 | 3,280,482,280 | 98.54% | 98.69% |
| MD-3 | Paired | 21,164,616 | 3,179,709,859 | 49.58% | 92.48% | 20,890,482 | 3,141,189,917 | 98.70% | 98.78% |
| PD-1 | Paired | 47,860,864 | 7,198,421,661 | 49.22% | 87.67% | 47,422,094 | 7,132,969,271 | 99.08% | 99.09% |
| PD-2 | Paired | 44,154,156 | 6,643,549,918 | 49.77% | 87.88% | 43,740,522 | 6,582,806,876 | 99.06% | 99.08% |
| PD-3 | Paired | 22,087,288 | 3,320,375,156 | 49.62% | 92.44% | 21,829,572 | 3,282,753,784 | 98.83% | 98.86% |
| PreD-1 | Paired | 35,169,052 | 5,285,304,125 | 48.66% | 86.17% | 34,755,490 | 5,225,092,523 | 98.82% | 98.86% |
| PreD-2 | Paired | 49,489,570 | 7,436,734,534 | 49.52% | 87.32% | 49,017,518 | 7,366,031,175 | 99.04% | 99.04% |
| PreD-3 | Paired | 18,593,682 | 2,794,657,282 | 49.74% | 91.37% | 18,370,312 | 2,762,811,734 | 98.79% | 98.86% |
