# Supplementary figures and images for: Transcriptomic and metabolomic profiles of Chinese citrus fly, Bactrocera minax (Diptera: Tephritidae), along with pupal development provide insight into diapause program
Source: PLoS One. 2017 Jul 12;12(7):e0181033. doi: 10.1371/journal.pone.0181033 (PMC5507520; doi:10.1371/journal.pone.0181033)

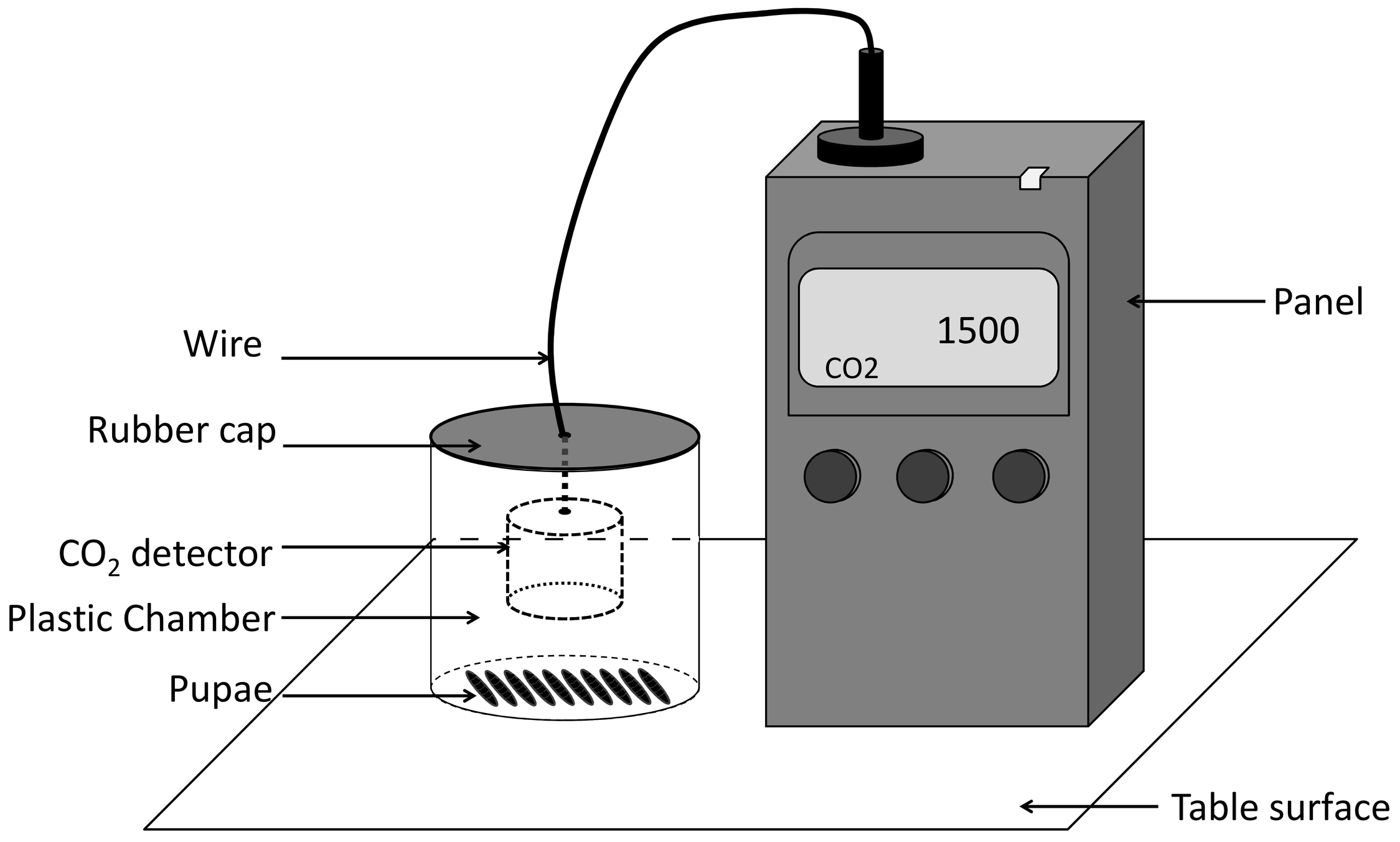

Supplement: S1 Fig — (TIF) [file pone.0181033.s003.tif]

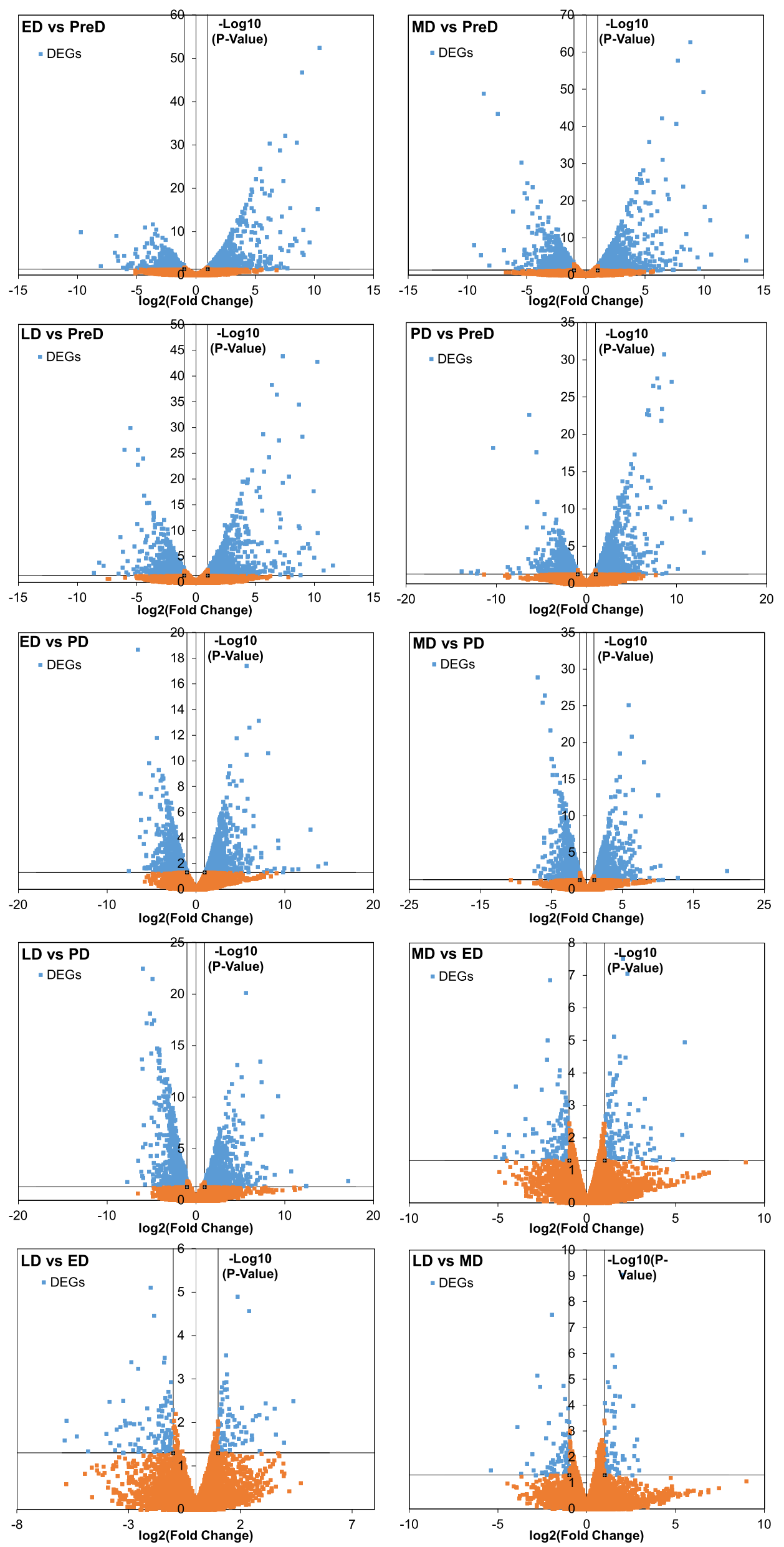

Supplement: S2 Fig — Blue spot, expression of fold change > 2 and P value < 0.05. Orange spot, no difference in expression. PreD, pre-diapause. ED, early-diapause. MD, middle-diapause. LD, late-diapause. PD, post-diapause. (TIF) [file pone.0181033.s004.tif]

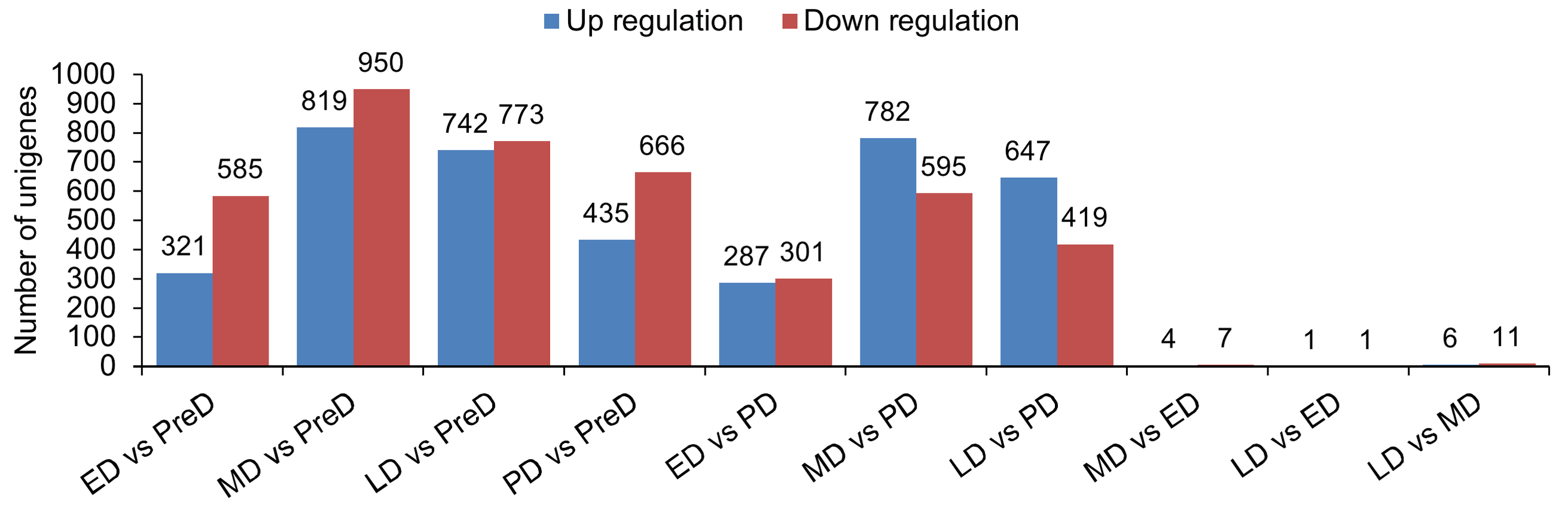

Supplement: S3 Fig — PreD, pre-diapause. ED, early-diapause. MD, middle-diapause. LD, late-diapause. PD, post-diapause. (TIF) [file pone.0181033.s005.tif]

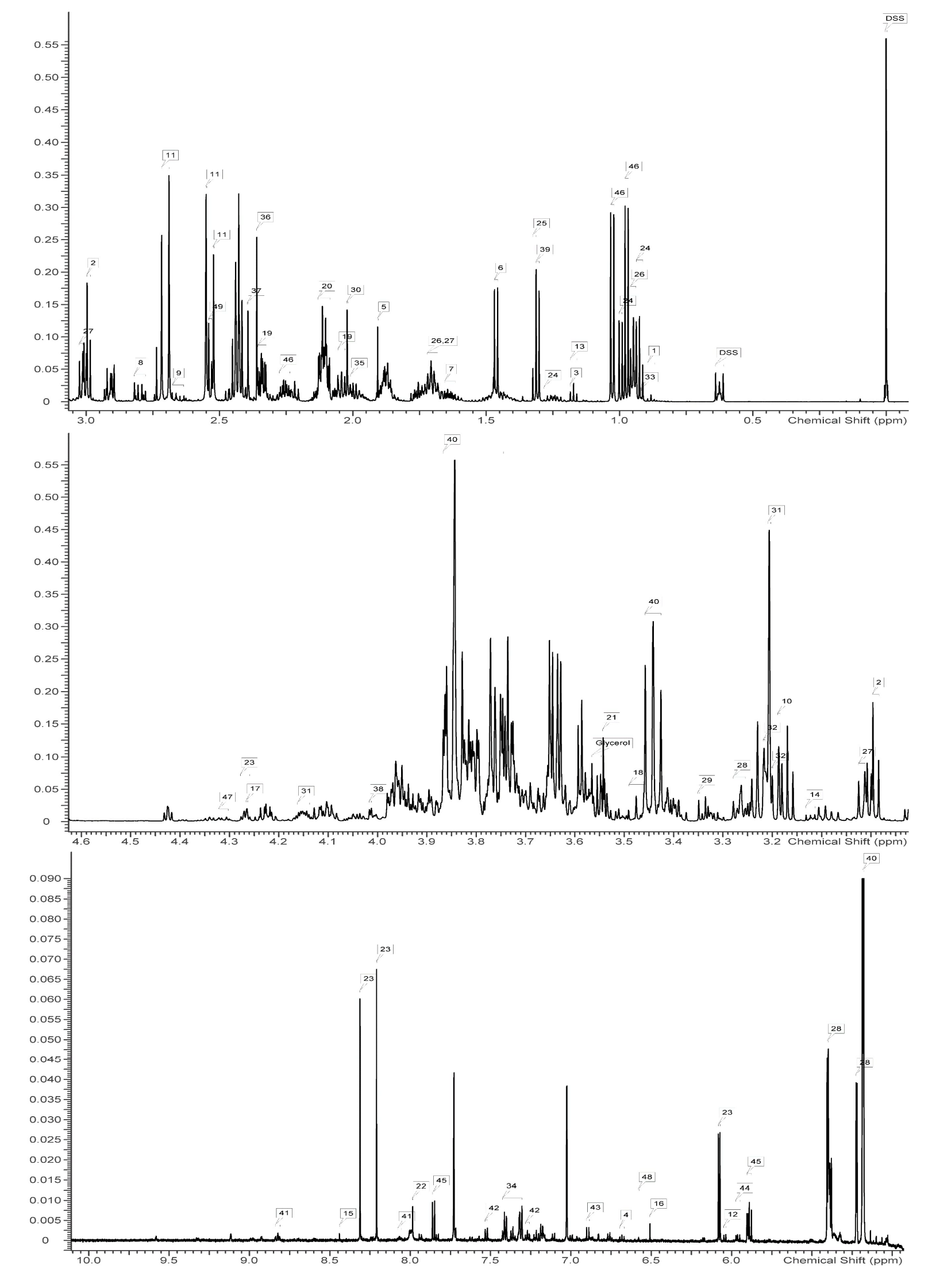

Supplement: S4 Fig — 1, 2-Hydroxybutyrate. 2, 2-Oxoglutarate. 3, 3-Aminoisobutyrate. 4, 3-Hydroxykynurenine. 5, Acetate. 6, Alanine. 7, Arginine. 8, Asparagine. 9, Aspartate. 10, Choline. 11, Citrate. 12, Cytidine. 13, Ethanol. 14, Ethanolamine. 15, Formate. 16, Fumarate. 17, Galactonate. 18, Glucose. 19, Glutamate. 20, Glutamine. 21, Glycine. 22, Guanosine. 23, Inosine. 24. Isoleucine. 25, Lactate. 26, Leucine. 27, Lysine. 28, Maltose. 29, Methanol. 30, N-Acetylglutamate. 31, O-Phosphocholine. 32, O-Phosphoethanolamine. 33, Pantothenate. 34, Phenylalanine. 35, Proline. 36, Pyruvate. 37, Succinate. 38, Threonate. 39, Threonine. 40, Trehalose. 41, Trigonelline. 42, Tryptophan. 43, Tyrosine. 44, UDP-N-Acetylglucosamine. 45, Uridine. 46, Valine. 47, sn-Glycero-3-phosphocholine. 48, trans-Aconitate. 49, β-Alanine. DSS, DSS Chemical Shape Indicator. (TIF) [file pone.0181033.s006.tif]

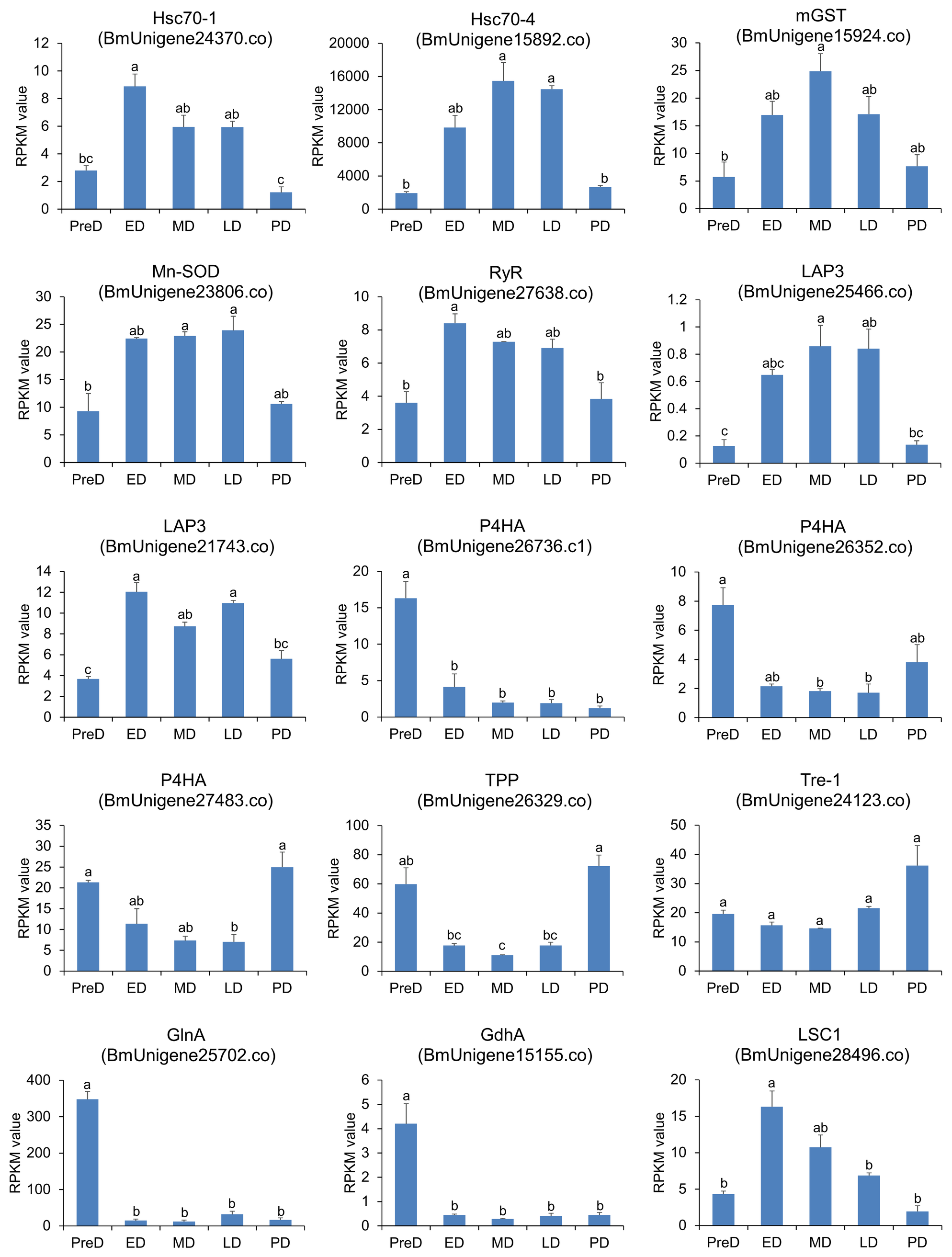

Supplement: S5 Fig — PreD, pre-diapause. ED, early-diapause. MD, middle-diapause. LD, late-diapause. PD, post-diapause. Hsc70, heat shock cognate protein 70. mGST, microsomal glutathione S transferase. Mn-SOD, Mn superoxide dismutase. RyR, ryanodine receptor. LAP3, cytosol aminopeptidases. P4HA, prolyl 4-hydroxylases. TPP, trehalose 6-phosphate phosphatase. Tre-1, trehalase-1. GlnA, glutamine synthetase. GdhA, glutamate dehydrogenase. LSC1, succinyl-CoA synthetase. (TIF) [file pone.0181033.s007.tif]
